# Industrial Use of Cell Wall Degrading Enzymes: The Fine Line Between Production Strategy and Economic Feasibility

**DOI:** 10.3389/fbioe.2020.00356

**Published:** 2020-04-29

**Authors:** Moira Giovannoni, Giovanna Gramegna, Manuel Benedetti, Benedetta Mattei

**Affiliations:** Department of Life, Health and Environmental Sciences, University of L’Aquila, L’Aquila, Italy

**Keywords:** cell wall degrading enzymes, biofactory, enzyme productivity, production cost, plant cell wall, microalgae, peptidoglycan, chitin

## Abstract

Cell Wall Degrading Enzymes (CWDEs) are a heterogeneous group of enzymes including glycosyl-hydrolases, oxidoreductases, lyases, and esterases. Microbes with degrading activities toward plant cell wall polysaccharides are the most relevant source of CWDEs for industrial applications. These organisms secrete a wide array of CWDEs in amounts strictly necessary for their own sustenance, nonetheless the production of CWDEs from wild type microbes can be increased at large-scale by using optimized fermentation strategies. In the last decades, advances in genetic engineering allowed the expression of recombinant CWDEs also in lab-domesticated organisms such as *E. coli*, yeasts and plants, dramatically increasing the available options for the large-scale production of CWDEs. The optimization of a CWDE-producing biofactory is a hard challenge that biotechnologists tackle by testing different expression strategies and expression-hosts. Although both the yield and production costs are critical factors to produce biomolecules at industrial scale, these parameters are often disregarded in basic research. This review presents the main characteristics and industrial applications of CWDEs directed toward the cell wall of plants, bacteria, fungi and microalgae. Different biofactories for CWDE expression are compared in order to highlight strengths and weaknesses of each production system and how these aspects impact the final enzyme cost and, consequently, the economic feasibility of using CWDEs for industrial applications.

## Introduction

The cell wall is a complex, selectively permeable layer surrounding the cell. It is found in archaea, bacteria, fungi, plants and algae. The most relevant functions of cell wall are to confer protection, structure and support to the cell; therefore, cell wall evolved to be highly resistant to a wide range of biotic and abiotic stresses. The cell wall composition varies greatly in organisms from different Kingdoms with further sub-differentiation amongst organisms of the same Kingdom. For example, plant cell walls are mainly composed of polysaccharides arranged in complex structures while the structural proteins are minor components; on the other hand, bacterial peptidoglycan is composed of polysaccharidic chains crosslinked by many peptide bridges. Heterogeneity and complexity of the cell wall are crucial, since it is the first line of defense against pathogens and predator attacks. In this regard, “attacking” organisms evolved different enzymes specialized in cell wall degradation, named as Cell Wall Degrading Enzymes (CWDEs). Intriguingly, the same CWDE can work both as attack and defense weapon, depending on the nature of the producing organism. For example, lysozyme produced by lytic bacteriophages hydrolyses peptidoglycan to favor the release of the viral progeny from the bacterial cell (Lavigne et al., 2004) while animals produce lysozyme to protect themselves against pathogenic bacterial infection (Ragland and Criss, 2017). Moreover, organisms evolved endogenous CWDEs to remodel their own cell wall structures during development. In general, endogenous CWDEs are characterized by milder degrading activities compared to those of exogenous nature and are therefore of lower industrial relevance. CWDEs are a highly heterogenous family including glycosyl-hydrolases (the most abundant class of enzymes), oxido reductases, lyases and esterases. The different CWDEs are classified in the Carbohydrate Active Enzymes (CAZy) database^1^, based on the sequence homology of their catalytic domains. Degradation of cell wall polysaccharides not only allows pathogens to penetrate inside the host cell, but also to release sugars for their own growth, thus sustaining the infection process. In the last decades, the use of cell wall polysaccharides or their derivatives for biofuel and chitosan production as well as the necessity to eliminate cell wall residues from products such as food, beverage, paper and textiles strongly boosted the use of CWDEs in many industrial applications (Benedetti et al., 2019a). Here, different CWDEs of industrial interest will be reviewed, indicating the most promising CWDE-expressing biofactories and the current methods employed for the large-scale production of CWDEs. Moreover, different CWDE-expressing biofactories, including wild type and transgenic organisms, will be analyzed in term of CWDE yield, enzyme productivity and production costs in order to highlight strengths and weaknesses of each expression system in the perspective of a possible large-scale application.

## Enzymatic Characteristics and Industrial Applications of CWDEs

In this section, the most studied CWDEs have been classified based on their target cell wall and substrate specificity. The main industrial applications for each CWDE category are summarized. In the last paragraph, the industrial potential of CWDEs from hyperthermophilic microorganisms is discussed.

### CWDEs Toward the Plant Cell Wall

The plant cell wall is composed mostly of carbohydrate-based polymers, i.e., cellulose, hemicelluloses and pectins. All the plant cells that are in developmental expansion have a primary cell wall that is constantly remodeled, composed of cellulose fibers embedded in a hemicellulose-pectin matrix (Burton et al., 2010). At the end of the plant cell development, i.e., once the cell has ceased to expand, a secondary layer composed by cellulose, hemicellulose and lignin is deposited close to the primary cell wall; the architecture of such assembly varies among different cell types, being optimized to perform cell-specific functions (Keegstra, 2010; Höfte and Voxeur, 2017; Zhong et al., 2019). Lignocellulose from agricultural feedstocks is formed by clusters of secondary cell walls; in terms of energetic potential, lignocellulose appears as a deposit of reducing power stored in its complex structure and composition. Phytopathogens as well as saprophytes secrete a wide array of CWDEs to open a breach in the plant cell wall; the degradation of this complex matrix into simple sugars significantly contribute to the global carbon cycle (Gibson et al., 2011; Choi et al., 2013; Glass et al., 2013). CWDEs are divided in many categories and sub-categories depending on their substrate specificities toward the diverse cell wall polysaccharides. The most important CWDEs directed toward the plant cell wall polysaccharides are:

#### CWDEs With Cellulolytic Activity

**FIGURE 1:**
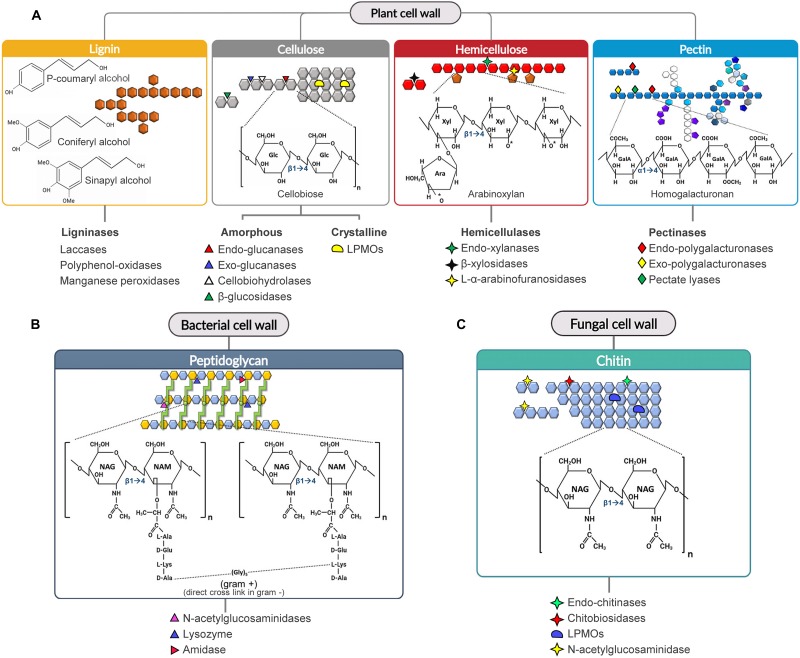
Substrate specificity of CWDEs toward the main cell wall polysaccharides. The cleavage sites of the major CWDEs involved in the degradation of lignocellulose **(A)**, peptidoglycan **(B)** and chitin **(C)** are shown. **(A)** Arabinoxylan and homogalacturonan are reported as examples of hemicellulose and pectin polysaccharides, **(B)** gram-positive peptidoglycan is reported as example of bacterial peptidoglycan. The cleavage sites of ligninases are not shown due to the complexity of the structure. In peptidoglycan, the green circle represents the dipeptide (D-Ala/L-Lys) connecting two peptide bridges (light green) from adjacent chains. [Ara: L-Arabinose, GalA: D-Galacturonic Acid, Glc: D-Glucose, NAG: *N*-acetylglucosamine, NAM: *N*-acetylmuramic acid, LPMO: lytic polysaccharide monooxygenase, Xyl: D-Xylose].

Cellulose is a β-1,4-homopolymer formed by repeated units of cellobiose, i.e., a disaccharide of D-glucose. Cellulose chains interact with each other to form fibrils and, at macroscale, fibers. Cellulolytic enzymes include glycosyl-hydrolases (i.e., cellulases) and oxidoreductases (i.e., lytic polysaccharide monooxygenases, LPMOs) (Singhania et al., 2013; Sandhu et al., 2018; Binod et al., 2019). Cellulases and LPMOs act in a synergistic way to degrade the amorphous and crystalline regions of cellulose, respectively (Dimarogona et al., 2012; Martínez et al., 2017; Obeng et al., 2017; Figure 1A). Cellulases degrade the amorphous region of cellulose by three main enzymatic activities: endo-glucanase, exo-glucanase (i.e., cellodextrinase and cellobiohydrolase) and β-glucosidase activities. Many cellulases (mainly endo- and cellobiohydrolases) are expressed as modular enzymes in which the catalytic domain (GH) is linked to a carbohydrate binding module (CBM). The presence of CBM is not essential for cellulase activity although it could increase both the recognition and binding of the substrate (Bayer et al., 1998; Payne et al., 2015; Sajith et al., 2016; Ahmed and Bibi, 2018).

Cellulolytic oxidases (LPMOs) are characterized by a broader substrate specificity with respect to cellulases, since they can also attack the crystalline region of cellulose. LPMOs catalyse the oxidative cleavage of crystalline cellulose thus exhibiting a synergistic action with hydrolytic enzymes (endoglucanases and cellobiohydrolases) (Villares et al., 2017; Hu et al., 2018). LPMO cleaves glycosidic linkages, leading to the formation of oxidized glucose units at C1 position (gluconic acid) and/or at the C4 position (4-ketoglucose) (Eibinger et al., 2014; Vaaje-Kolstad et al., 2017; Valenzuela et al., 2019). The copper-containing active site of LPMO must be reduced after each reaction in order to guarantee the enzyme turnover and different reducing mechanisms that can assist LPMO recycling have been identified. Electrons can be restored to LPMO through the following mechanisms: (i) cellobiose oxidation by cellobiose dehydrogenase (Loose et al., 2016; Laurent et al., 2019), (ii) monolignol oxidation upon lignin degradation (Brenelli et al., 2018) and (iii) H_2_O_2_-mediated reduction (Müller et al., 2018; Eijsink et al., 2019). The latter mechanism was elucidated for the first time in 2017, paving the way to novel strategies for improving cellulose degradation.

#### CWDEs With Hemicellulolytic Activity

Hemicellulose is a branched and highly heterogenous polysaccharide composed of pentoses (e.g., xylose and arabinose), hexoses (e.g., mannose, glucose and galactose) and acidic sugars (e.g., galacturonic and glucuronic acid) (Scheller and Ulvskov, 2010; Zhao et al., 2012). Due to its high complexity, many hemicellulolytic enzymes are required for the efficient degradation of hemicellulose. Efficient degradation of xylan, i.e., the most abundant hemicellulose in agricultural wastes, is particularly relevant to prevent the formation of by-products with inhibitory activity toward cellobiohydrolases (Baumann et al., 2011; Momeni et al., 2015; Malgas et al., 2019) and downstream processes such as yeast fermentation to produce bioethanol (Jönsson and Martín, 2016). Unlike cellulose, hemicellulose is mainly hydrolysed by glycosyl-hydrolases. The most used in industrial applications are endo-acting CWDEs such as xylanases, mannanases and galactanase, and exo-acting CWDEs such as β-galactosidases, β-xylosidase, β-mannosidase and L-α-arabinofuranosidase (Figure 1A).

#### CWDEs With Pectinolytic Activity

Pectin is a branched acidic α-(1,4)-polysaccharide consisting mainly of D-galacturonic acid and of various proportions of other sugars including L-rhamnose, L-arabinose, D-galactose and D-xylose. Pectin is composed of different domains, i.e., homogalacturonan, xylogalacturonanan, rhamnogalacturonan I and rhamnogalacturonan II, covalently linked to each other (Ridley et al., 2001; Caffall and Mohnen, 2009). In homogalacturonan the D-galacturonic acid units can be methyl-esterified (at C6) and/or O-acetylated (at C2 and/or C3 hydroxyl groups). Pectin is degraded by microbial glycosyl-hydrolases (i.e., endo-polygalacturonases and exo-polygalacturonases) and lyases (i.e., pectate lyases) (Figure 1A).

#### CWDEs With Ligninase Activity

Lignin is a complex phenolic polymer derived from phenylpropanoid monolignols, that confers strength and rigidity to the plant cell wall (Figure 1A); hence, its degradation can greatly improve the digestibility of lignocellulosic biomass. Lignin occludes the cellulose-hemicellulose assembly thereby increasing its recalcitrance to enzymatic hydrolysis, due to its hydrophobic nature and to the intrinsic characteristic of lignin to irreversibly adsorb to the CBM of CWDEs, thus poisoning the enzymes (Gao et al., 2014; Li et al., 2016). Lignin degradation is mainly catalyzed by ligninases, a general term that includes laccases, polyphenol-oxidases and (manganese)-peroxidases (Figure 1A).

#### Industrial Applications of CWDEs Toward the Plant Cell Wall

**FIGURE 2:**
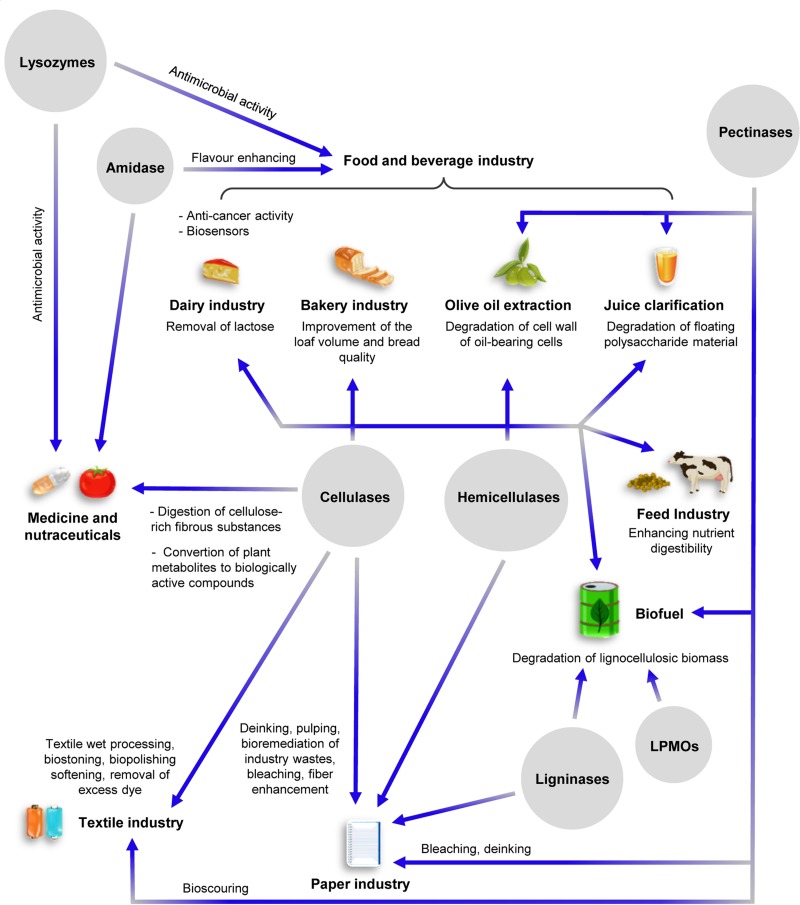
Schematic overview of the major industrial applications of CWDEs. Specific roles of the various CWDEs in the different industrial sectors are shown. The intersecting lines between cellulases and hemicellulases indicate common industrial applications.

Sugars from lignocellulosic wastes can be exploited as carbon source for the production of second generation biofuels (e.g., bioethanol by yeast fermentation) and biogas (methane by anaerobic digestion of methanogens) (Benedetti et al., 2019a; Herrero Garcia et al., 2019); however, the low efficiency of lignocellulose degradation by CWDEs, together with the high cost of commercial CWDE-based blends, make the entire process not competitive over chemical and physico-chemical treatments (Saini et al., 2015). Although chemical treatments are polluting, their use allows to reach higher degradation efficiencies at reduced cost compared to biological methods. In this perspective, the optimization of CWDE-expressing biofactories is mandatory to increase the sustainability of biological treatment of biomasses. Enzymatic degradation of lignocellulose may be increased by exploiting the synergistic action between different CWDEs. Many efforts have been spent by companies aiming at identifying the best-performing CWDE-combinations with maximum hydrolysis yield and minimum enzyme loading; as a consequence, many commercial enzyme-based products [e.g., Cellic^®^CTech (Novozymes A/S, Bagsværd, Denmark), MiaMethan^®^ ProCut (MIAVIT GmbH, Essen, Germany)] are protected by strict commercial laws so that their composition and production process are confidential. In addition to the biofuel sector, endo-glucanases, exo-glucanases and β-glucosidases are widely used also in food and feed processing, bakery, textile, cellulose pulping and paper industry as well as in the production of dietary supplements and nutraceuticals (Jayasekara and Ratnayake, 2019; Figure 2). Notably, β-glucosidases can be also used for removing lactose from dairy products due to their broad substrate specificity (Singh et al., 2016). Endo-xylanase and xylosidases are used in feed and bakery industry, while β-galactosidase and α-L-Arabinofuranosidase are employed in food processing (Figure 2). Pectinolytic enzymes are widely used in several industrial applications such as food and beverage processing, olive oil extraction, recycling of wastepaper, textile industry and wine/tea processing (Garg et al., 2016) (Figure 2). Ligninases have a great potential for industrial applications, although mechanisms underlying lignin degradation are largely still unknown. Currently, the use of ligninases is mainly restricted to the paper industry for the bleaching of cellulose pulp (Figure 2).

### CWDEs Toward the Bacterial Cell Wall

The bacterial cell wall is mainly composed of peptidoglycan, a heteropolymer consisting of amino sugars and oligopeptides. The backbone chain is formed by alternating residues of β-1,4(linked) N-acetylglucosamine (NAG) and N-acetylmuramic acid (NAM) (Vollmer et al., 2008; Dörr et al., 2019); NAM residues from adjacent chains, in turn, are interconnected by peptide bridges of variable length, ranging from three to five amino acids. Although the peptidoglycan composition is conserved among bacterial species, the overall cell wall architecture varies between Gram-positive, Gram-negative and acid-fast bacteria (Desvaux et al., 2018). The degradation of peptidoglycan is performed by amidases and glycosidases (Figure 1B; Alcorlo et al., 2017; Vermassen et al., 2019).

#### CWDEs With Amidase Activity

Amidase (N-acetylmuramoyl-L-alanine amidases, NALAAs) cleaves the peptide bridge of peptidoglycan by hydrolysing the amide bond connecting the first amino acid (L-alanine) to the NAM residue (Höltje, 1995; Vollmer et al., 2008; Irazoki et al., 2019). Amidases are grouped based on their catalytic site and substrate specificity (i.e., short, mid-chain amides, arylamides, α-aminoamides and α-hydroxyamides). These enzymes are associated in compact multimeric structures and are resistant both to high temperature and alkaline conditions (Sharma et al., 2009).

#### CWDEs With Glycosidase Activity

Glycosidases with degrading activity toward peptidoglycan comprise N-acetyl-glucosaminidase (NAGases) and lysozyme. NAGase hydrolyses the glycosidic bond between the N-acetyl-β-D-glucosamine residue and the closer monosaccharide; this type of bond is present in various oligosaccharides, chitin and N-glycans (Karamanos, 1997). Lysozyme hydrolyses the β-1,4 linkage between NAM and NAG residues in the peptidoglycan backbone (Herlihey and Clarke, 2017).

#### Industrial Applications of CWDEs Toward the Bacterial Cell Wall

The industrial applications of amidases ranges from biomedical field (clinical diagnostics and health monitoring) to the food industry as flavor enhancers (Nandakumar et al., 2003; Ghonemy, 2014; Kumar et al., 2014; Sathish et al., 2018). NAGases are mainly employed in organic chemistry to produce synthetic oligosaccharides (Scigelova and Crout, 1999). Lysozyme has many industrial applications ranging from food processing to medicine. It is used as antibacterial agent and pharmacological adjuvant for its capability to hydrolyse bacterial peptidoglycan and is also used as preservative in food processing with the identification code E1105. Moreover, lysozyme is used in combination with chitinases and cellulases to degrade chitin into chitosan, i.e., a polysaccharide formed by the repeated unit β-1,4-D-glucosaminyl-N-acetyl-D-glucosamine (Pillai et al., 2009). Chitosan and its derivatives are employed in the production of materials for medical surgery, for tissue engineering and drug delivery (Rinaudo, 2006; Zhang et al., 2010). Recently, chitosan was also used as component of nutraceuticals due to its ability to stimulate the immune system (Fong and Hoemann, 2018; Abdel-Tawwab et al., 2019; Figure 2).

### CWDEs Toward the Fungal Cell Wall

Fungal cell wall is a two-layered structure, with a hydrophobic and rigid inner layer, consisting of a branched β-1,3(linked) gluco-polysaccharide cross-linked to small portions of chitin (Kang et al., 2018); this layer is covalently linked to galactomannan and other branched glucans that, together, form the outer layer of the fungal cell wall (Gow et al., 2017; Kang et al., 2018). The outer layer is more hydrophilic and flexible than the inner layer due to the lack of chitin. Chitin is a homopolymer composed of β-1,4(linked) NAG residues. Notably, chitin is also a major component of the exoskeletons of arthropods, crustaceous shells, mollusc radula and cell walls of several microalgae species (Cauchie, 2002). Chitin naturally occurs in three different crystalline structures depending on the orientation of the constituting microfibrils. α-type and β-type chitin are characterized by antiparallel and parallel chains, respectively; γ-type chitin is a mixture of α-type and β-type chitin (Rudall and Kenchington, 1973). Chitin degradation is performed by chitinases, i.e., glycosyl-hydrolases that cleave the glycosidic bond between the NAG residues of chitin (Figure 1C).

#### CWDEs With Chitinolytic Activity

Chitinases are divided in endo-chitinases and exo-chitinases depending on their mode of action. Endo-chitinases hydrolyze both α- and β-type chitin releasing chito-oligosaccharides with different degree of polymerization, including small-size oligosaccharides such as di-acetylchitobiose, chitotriose, and chitotetraose. Exo-chitinases comprise chitobiosidases and β-1,4-N-acetylglucosaminidases. Chitobiosidase acts on the non-reducing end of the chitin chain by releasing di-acetylchitobiose (Harman, 1993), while β-1,4-N-acetylglucosaminidase cleaves chito-oligomers and chitobiose into NAG monomers.

#### Other CWDEs With Degrading Activity Toward Chitin

Other CWDEs are involved in chitin degradation by synergistically acting with chitinases, namely cellulases and LPMOs (see section “CWDEs Toward the Plant Cell Wall” for further details). Some LPMOs orthologs are more active in degrading crystalline chitin rather than cellulose (Vaaje-Kolstad et al., 2010; Bissaro et al., 2018).

#### Industrial Applications of CWDEs Toward the Fungal Cell Wall

Degradation of chitin by chitinases releases oligomers with different degree of polymerization that have applications in the biomedical and nutraceutical fields. In addition to chitin hydrolysis, chitinases are attracting great interest because of their potential application in agriculture, cosmetics and wastewater treatment (Synowiecki and Al-Khateeb, 2003; Park et al., 2011; Stoykov et al., 2015; Figure 2). Although chitin is the second most abundant biopolymer after cellulose, the use of chitinases in industrial processes is limited mainly by the lack of efficient chitinase-expressing biofactories (Stoykov et al., 2015).

### CWDEs Toward the Microalgal Cell Wall

Microalgae are found in a wide range of habitats and have adapted to a variety of environmental conditions by evolving a great genetic diversity, making them a precious source of interesting and useful metabolites (Benedetti et al., 2018a). However, the extraction of metabolites from microalgae represents one of the major bottlenecks limiting their potential in industrial applications. Enzymatic treatment is much less efficient than physical rupture, pointing to the necessity of developing CWDE blends effective in the degradation of the microalgal cell wall. It is worth noting that a univocal structural model of the microalgal cell wall cannot be established likely due to the broad adaptive diversification of microalgae. For example, the cell wall of the unicellular green alga *Chlamydomonas reinhardtii* is formed by seven layers of hydroxyproline-rich glycoproteins (Imam et al., 1985) while the cell wall of the marine microalga *Nannochloropsis gaditana* is composed by polysaccharides, i.e., cellulose and algaenan (Scholz et al., 2014). In this paragraph, we will focus on the cell wall of *Chlorella vulgaris*, one of the most employed oleaginous microalgae with applications ranging from the biofuel sector (i.e., production of biodiesel through lipid transesterification) to nutraceutical (Benedetti et al., 2018a; Dall’Osto et al., 2019). Although *C. vulgaris* is a well-studied organism compared to other microalgae species, the degradation of its cell wall is still a high hurdle. Preliminary studies demonstrated that the cell wall of *C. vulgaris* and other related microalgae species had rigid wall components embedded within a more plastic polymeric matrix. The acid-hydrolysis of this polymeric matrix revealed the presence of acid sugars, rhamnose, arabinose, fucose, xylose, mannose, galactose and glucose (Takeda, 1991). Subsequently, Gerken and collaborators showed the cell wall of *C. vulgaris* is constituted by a heterogeneous bilayer matrix; the inner layer is mainly composed of polysaccharides such as cellulose and pectin, while the external one is composed by a robust chitin-like glucan (Gerken et al., 2013).

#### CWDEs With Degrading Activity Toward *C. vulgaris*

Lysozyme from hen egg-white is the most effective CWDEs in degrading the cell wall of this microalga, followed by the endo-chitinase from *Streptomyces griseus*, the sulfatases from *Helix pomatia*, β-glucuronidase and laminarinase (Gerken et al., 2013; Kumar et al., 2018). Sulfatases belong to the esterase class catalyzing the hydrolysis of sulfate esters in steroids, carbohydrates and proteins (Parenti et al., 1997). The sulfatases from *H. pomatia* are divided in H1- and H2-type sulfatase, depending on their substrate specificity. β-glucuronidase is a glycosyl-hydrolase catalyzing the hydrolysis of β-D-glucuronic acid residues from the non-reducing end of mucopolysaccharides (Sinnot, 1998), while laminarinase catalyzes the endo-hydrolysis of 1,3- or 1,4-linkages in β-D-glucans when the glucose residue involved in the linkage is substituted at C3 position (Salyers et al., 1977). At present, the enzymatic degradation of *C. vulgaris* requires huge amounts of CWDEs making the process not competitive at industrial scale (Gerken et al., 2013; Kumar et al., 2018). In conclusion, the many different (and apparently unrelated) enzymatic activities used to degrade *C. vulgaris* reflect on one hand the hybrid nature of its cell wall, and on the other hand point to the necessity of further investigations.

### CWDEs From Hyperthermophiles

Cell wall degrading enzymes from hyperthermophilic microbes (HCWDEs) represent a category of high industrial interest due to their peculiar enzymatic characteristics. These enzymes are also known as “Hot Extremozymes” since they are active at temperatures ranging from 70 to 100°C (Sarmiento et al., 2015). The high temperature required for optimal activity and stability of HCWDEs allows faster and more effective reactions (Yeoman et al., 2010). Moreover, elevated temperature prevents undesired growth of contaminating microbes during the catalysis, thus improving the conversion yield of cell wall polysaccharides into simple sugars. Proteinaceous CWDE-inhibitors, that are widely distributed in the plant cell wall as a defense mechanism (York et al., 2004; Juge, 2006; Mohammadzadeh et al., 2012; Kalunke et al., 2015), are inactivated by high temperature, thus avoiding interference with the enzymatic reaction. Another important feature of HCWDEs is protein stability that allows prolonged storage at room temperature and resistance to harsh conditions, e.g., the presence of aggressive chemicals, anionic/non-ionic detergents and extreme pH (Benedetti et al., 2019b), that can be exploited to deconstruct more efficiently cell wall recalcitrant material. Stability of HCWDEs also allows an efficient enzyme recycling over time, thus reducing the total enzyme loading in industrial practices. However, maintaining industrial processes at high temperature for a long time requires a great expense of energy, therefore a further step toward sustainability may imply the use of HCWDEs in industrial plants with excess heat that can be recycled in order to limit the additional heating cost. Up to now, not all the CWD-activities toward plant cell wall polysaccharides are available in their respective hyper-thermostable version. In particular, while hyper-thermostable orthologs have been isolated for mesophilic cellulases, hemicellulases and ligninases, the exo-polygalacturonases from *Thermotoga maritima* and *Caldicellulosiruptor bescii* (Kluskens et al., 2005; Chen et al., 2014) are the only pectinases isolated so far, and neither endo-polygalacturonases nor pectate lyases of hyperthermophilic nature have been identified yet. Similarly, LPMOs from the thermophilic bacterium *Thermobifida fusca* are the only available option for the degradation of crystalline cellulose at mid-high temperature (Moser et al., 2008). Other carbohydrate active enzymes with important industrial applications are amylases, employed in starch conversion, biofuel production, brewing, bakery, textile, detergent and paper industry. Well-known α-amylase producers are bacteria belonging to the genus Bacillus such as *B. subtilis, B. licheniformis*, and *B. amyloliquefaciens* (Jujjavarapu and Dhagat, 2019), while β-amylases are mainly obtained by plants such as barley (*Hordeum vulgare*) and sweet potato (*Ipomoea batatas*) (Saini et al., 2017). Unlike α-amylases from Bacillus species, β-amylases are less resistant to high temperature; in this regard, the use of a hyper-thermostable β-amylase may be a cost-effective choice for reducing the amount of enzyme used during the catalysis; to date, the only β-amylase of hyperthermophilic nature with proven activity was identified from *Clostridium thermosulfurogenes* (Nipkow et al., 1989).

Other HCWDEs of industrial interest are those degrading fungal and bacterial cell wall polysaccharides. A highly thermostable chitinase was isolated from *Pyrococcus furiosus* (Oku and Ishikawa, 2006); this enzyme showed marked degrading activity toward both the amorphous and β-type chitin, while it was less active toward α-type chitin. Noteworthy, highly thermostable lysozymes were also identified; they were isolated from hyperthermophilic bacteriophages such as the Pseudomonas phage φ *KMV* (Lavigne et al., 2004); the substrate specificity of thermostable lysozymes is not comparable to that of egg-white lysozyme commonly used in food processing, thereby precluding their exploitation in this field.

The industrial use of HCWDEs has been so far limited by the fact that they cannot be efficiently produced at large-scale by wild-type microbes. Production of recombinant HCWDEs may be carried out in *E. coli* by high cell-density fermentation. As an alternative expression system, the last advances in plant biotechnology allowed the large-scale production of recombinant HCWDEs also in tobacco plants (Castiglia et al., 2016; Schmidt et al., 2019) (*see* section “Yield and Cost Analysis of Plant CWDE-Producing Biofactory”). In Table 1, a selection of CWDEs with both consolidated and potential use in industrial applications is proposed. According to this selection, the use of HCWDEs is suggested for biofuel production rather than food processing since the high temperature of action may alter the organoleptic properties of food and beverages. The techno-economic aspects of different CWDE-expressing biofactories are discussed in section “YIELD AND COST ANALYSIS OF ENZYMES FROM DIFFERENT CWDE-PRODUCING BIOFACTORIES.”

**TABLE 1:** Industrial application of CWDEs.

Uniprot code	Source	Catalytic domain	Enzyme	Enzyme features				Cell-wall products (intermediate-, end-product)	References

				Reference substrate	T_*opt*_ (°C)	pH_*opt*_	Activity μmol/min*mg		
P96492	*T. neapolitana*	GH12	Endo-1,4-β-glucanase	PASC	106	6.3	38.5	cellodextrins, cellobiose	Bok et al., 1998
O08428					95	6.0	9.45	cellodextrins, cellobiose	
P10474	*C. saccharolyticus*	GH5	Cellobiohydrolase	*p*NPC	80	5.5	0.62	Cellobiose	Park et al., 2011
Q51723	*P. furiosus*	GH1	β-glucosidase	cellobiose	104	5.0	470	D-glucose	Kengen et al., 1993
Q60042	*T. neapolitana*	GH10	Endo-1,4-β-xylanase	oat xylan	103	5.5	111.3	xylo-oligomers, D-xylose	Zverlov et al., 1996
B9K9B3		GH53	endo-β-1,4-galactanase	galactan	90	6.5	159*	galacto-oligomers, D-galactose	Benedetti et al., 2019a
B9KC33		GH51	α-L-arabinofuranosidase	*p*NPAF	90	5.0	147*	L-arabinose	
Q9RIK7		GH5	β-mannosidase	mannobiose	92	7.0	56	D-mannose	Parker et al., 2001
Q9RIK9	*T. maritima*		β-mannanase	locust bean gum	92	7.1	3.8	manno-oligomers, mannobiose	
Q72HW2	*T. termophilus*	AA1	Laccase	ABTS	92	4.5	30.1*	Monolignols	Miyazaki, 2005
Q47QG3	*T. fusca*	AA10	LPMO	Filter paper	50	6.0	ND	C1/C4 oxidized cellodextrins	Moser et al., 2008
A9XK88	*M. thermophilum*	AA3/AA8	CDH	cellobiose	63	5.5	19.8*	C1 oxidized cellobiose	Zámockẏ et al., 2008
B9MNB8	*C. bescii*	GH28	1,4-α-galacturonidase	PGA	72	5.2	384.6	D-galacturonic acid	Chen et al., 2014
Q9WYR8	*T. maritima*		Exo-galacturonosidase		80	6.4	1170		Kluskens et al., 2005
Q6CZT4	*E. carotovora*	PL-C	Pectate lyase	PGA	40	8.3	1600	unsaturated pectin-oligomers	Lei et al., 1987
Q07181	*G. fujikuroi*	GH28	Polygalacturonase		ND	5.0	500	pectin-oligomers digalacturonic acid D-galacturonic acid	Federici et al., 2001
P26509	*E. carotovora*				40	5.5	ND		Saarilahti et al., 1990
P26214	***A. niger***				ND	4.1	2000		Benen et al., 1996
G0RUP7	***T. reesei***	GH11	Endo-1,4-β-xylanase	glucuronoxylan	60	6.0	ND	xylo-oligomers, D-xylose	Saarelainen et al., 1993
P62694		GH7	Exo-glucanase	DNP-Lac	50	5.0	0.72	D-glucose	Becker et al., 2001
Q2F8H3			Endo-glucanase	CMC	55	5.0	220.2	cellodextrins, cellobiose	Samanta et al., 2012
A0A223GCX3	***A. niger***	GH11	Endo-1,4-β-xylanase	oat spelts xylan	50	5.5	3881	xylo-oligomers, D-xylose	Levasseur et al., 2005
ND	***B. licheniformis***	ND	α-amylase	soluble starch	90	9.0	77.1*	Maltodextrins, maltotriose	Krishnan and Chandra, 1983
P29761	***Clostridium sp.***	GH15	Gluco-amylase	Malto-heptaose	ND	4.5	66.3*	D-glucose	Ohnishi et al., 1992
Q7X3S6	***B. licheniformis***	GH5	Endoglucanase	CMC	65	ND	ND	cellodextrins, cellobiose	Liu et al., 2004
D1L8C5		GH9							
Q7X4S4		GH12							
P50401	*C. fimi*	GH6	Exo-glucanase		37	7.0	0.04*	cellobiose, D-glucose	Meinke et al., 1994
P00722	*E. coli*	GH2	β-galactosidase	*o*NPG	ND	7.0	256.9*	D-galactose	Sutendra et al., 2007
Q45071	***B. subtilis***	GH43	Arabinofuranohydrolase	wheat bran	45	5.6	3.2	L-arabinose	Bourgois et al., 2007
O50152	*S. griseus*	GH19	Endochitinase	glycol chitin	ND	6.0	10100	chito-oligomers	Itoh et al., 2002
P07254	*S. marcescens*	GH18	Chitobiosidase	MU-chi2	ND		13.3	Chitobiose	Brurberg et al., 1994
ND	*T. emersonii*	ND	N-acetylglucosaminidase	MU-NAG	75	5.0	534.3*	*N*-acetylglucosamine	O’Connell et al., 2008
P00698	*G. gallus* **(egg)**	GH22	Lysozyme C	*M. luteus* suspension	37	6.2	70400#	Peptidoglycan oligomers	Chiang et al., 1993
P61626	*H. sapiens*	GH22			40	6.5	201526#		Huang et al., 2002a

Different CWDEs with enzymatic characteristics of industrial interest are shown. The optimal temperature and pH, the specific activity expressed as (μmol min^–1^ mg enzyme^–1^) toward a reference substrate together with the main intermediate and end-products from cell wall polysaccharides are indicated for each CWDE. Organisms with a consolidated use in industrial field are in bold. CWDEs involved in: [P96492-Q51723], amorphous cellulose degradation; [Q60042-Q9RIK9], hemicellulose degradation; [Q72HW2-A9XK88], lignin and crystalline cellulose degradation; [B9MNB8, Q9WYR8], pectin degradation; [Q6CZT4-P26214], juice clarification and olive oil extraction; [G0RUP7- P29761], bakery-product improvement; [G0RUP7-A0A223GCX3], animal feed processing; [Q7X3S6-Q45071], digestive supplements; [P00698-P61626], food preservative; [O50152-P61626], chitosan production. CWDEs are identified by their UNIPROT code whenever possible (www.uniprot.org). *, values calculated from the available data in the original manuscript. #, values are in Shugar unit: one Shugar unit is defined as the amount of enzyme that will cause a decrease in absorbance of 0.001 at 450 nm per min due to lysis of *M. luteus* at 25°C, pH 6.2. Annotation of conserved domains is in accordance with the CAZy database (http://www.cazy.org): AA, Auxiliary Activity; GH, Glycoside Hydrolase; PL, Polysaccharide Lyase; ND, not determined. [ABTS, 2,2′-azino-bis(3- ethylbenzothiazoline-6-sulfonic acid); CDH, cellobiose dehydrogenase; CMC, carboxymethylcellulose; DNP-Lac, 3,4-dinitrophenyl-β-D-lactoside; LPMO, Lytic Polysaccharide Monooxygenase; MU-NAG, 4-Methylumbelliferyl-N-acetylglucosaminide; MU-chi2, 4-Methylumbelliferyl-N,N′-diacetyl-β-D-chitobioside; *o*PNG, ortho-Nitrophenyl-β-D-galactoside; PASC, Phosphoric Acid Swollen Cellulose, PGA, polygalacturonic acid; *p*NPAF, 4-Nitrophenyl-α-L-arabinofuranoside; *p*NPC, para-Nitrophenyl-β-D-cellobioside].

## Large-Scale Production of CWDEs

Cell wall degrading enzymes can be produced at large-scale through different expression strategies. Nowadays, microbial fermentation still plays a prominent role in the production of commercial enzymes. However, although both endogenous and recombinant CWDEs may be expressed through microbial fermentation, recent advancement in genetic engineering of land-plants and microalgae increased the spectrum of potential expressing hosts. In this section, the biotechnological aspects of conventional and novel CWDE-expressing biofactories are discussed (Table 2).

**TABLE 2:** Biotechnological aspects of different CWDE-expressing biofactories.

Organisms	Type of expression		Production method			Potential CWDE side effects	Post-translational modifications		CWDE Secretion

	endogenous CWDE Mix	HE	Type	Annual biomass productivity t (ha*y)^–1^	Biomass cost (€ kg DW^–1^)		Disulphide bridges	Glycosylation	
Bacteria e.g., Bacillus, Clostridium	YES	NO	SF SSF	NA	NA	NA	NA	NA	NA
Filamentous fungi e.g., Trichoderma, Aspergillus									
*E. coli*	NA	N	HDF			YES	NO	NO	YES**
Yeasts e.g., Kluyveromyces, Pichia							YES	YES*	YES
Plants *N. tabacum*		C	F	8–8.1^*a*^	2^*a*^			NO	NA
		N						YES	
Microalgae e.g., *C. reinhardtii*		C N	PBR	60^*b*^	3.8^*b*^			NO YES	NO YES
									

Bacteria and filamentous fungi with cell-wall degrading activities can produce mixtures of endogenous CWDEs (CWDE MIX) by submerged (SF) and solid-state fermentation (SSF). The desired CWDE can be expressed in *E. coli* and yeast by high-cell density fermentation (HDF) through heterologous expression (HE). Alternatively, transgenic plants and microalgae can produce recombinant CWDEs in open field (F) and closed photobioreactors (PBR), respectively, by both nuclear (N) and chloroplast expression (C). Biomass productivity and cost are key factors for those biofactories that combine the production of CWDEs and valuable biomass, i.e., plant and microalgae. *, hyperglycosylation may occur. **, secretion possible but challenging. NA, not applicable. [C, chloroplast transgene expression; F, open field cultivation; HDF, high-cell density fermentation; N, nuclear transgene expression; PBR, photobioreactor cultivation; SF, submerged fermentation; SSF, solid-state fermentation]. ^*a*^Schmidt et al., 2019. *Nat. Plants* 5, 715–721. ^*b*^Tredici et al., 2016. *Algal Res* 19, 253–263.

### Microorganisms as CWDE-Producing Biofactories

Cellulolytic and pectinolytic microbes are mainly cultivated by submerged and solid-state fermentation. In submerged fermentation, bacteria and filamentous fungi are grown in liquid medium containing nutrients, macro and micro-elements under sterile conditions and continuous oxygen supplementation. The most appropriate carbon source is selected depending on the type of expressed CWDE, e.g., glucose and galacturonic acid for optimal production of cellulases and polygalacturonases, respectively. Submerged fermentation is usually operated in fed-batch or continuous culture. In fed-batch cultures, enzyme production occurs mainly during the first phase of biomass growth. Subsequently, the growth rate in the culture is maintained by adding nutrients at different time-points, thus reducing the risk of overflow metabolism. On the opposite end, continuous fermentation requires an open growth system where sterilized solutions of nutrients are added *in continuum* to the bioreactor at the same rate at which the fermented medium is recovered from the system. This procedure results in a steady-rate production of the fermentation broth. In order to maintain the fermentation as efficient as possible, parameters such as temperature, pH, oxygen and carbon dioxide levels must be continually monitored and adjusted.

Solid-state fermentation is used as an alternative to submerged fermentation. Solid-state fermentation occurs in the absence of free water, can usually reach greater volumetric productivity than submerged fermentation and is also characterized by an easier downstream process. In general, the solid-state fermentation involves a solid matrix (e.g., rice bran, wheat bran, steam exploded agricultural scraps) that is used as feed by the selected microbe. The substrate matrix is maintained for days at controlled temperature under constant or intermittent rotation allowing the growth of filamentous fungi under conditions that resemble their natural environment. Air flow rate must be monitored since it affects both the humidity and oxygen levels of the entire fermentation process. The use of sterilization procedures is not mandatory for solid-state fermentation except for the sterilization of the substrate at the beginning of the process. In general, solid-state fermentation has several advantages over submerged fermentation, such as the lower consumption of water and electricity, a lower waste efflux and a more concentrated enzymatic product (Zhuang et al., 2007). These three parameters impact the final cost of the enzyme-based product that will be cheaper than that obtained *via* submerged fermentation. At the end of the fermentation process, the CWDEs are separated from the residual materials by micro- and ultra-filtration procedures (Machado de Castro et al., 2010; Ferreira et al., 2018). Subsequently, the enzyme preparation will be concentrated at the desired level and stabilizing agents will be supplied to the preparation in order to increase the shelf-life of the enzyme-based product. Notably, if a high-value product is required as in the case of CWDEs for use in food processing and biomedical field, further purification procedures will be necessary in order to eliminate residual contaminants, thus increasing the final cost of the enzyme.

Pectinases, cellulases, xylanases, and α-amylases are produced by microbial fermentation. By adjusting the carbon source used for inducing CWDE production, different enzymes can be secreted in the same fermentation broth; e.g., *Aspergillus awamori*, a fungus with GRAS (Generally Recognized As Safe) designation, secreted simultaneously cellulases, xylanases and α-amylases when fed with the babassu cake, i.e., the residual material from babassu palm (*Attalea speciosa*) upon oil extraction (Machado de Castro et al., 2010).

One of the most relevant fungi for cellulase production is *Trichoderma reesei.* This filamentous fungus is employed as cellulase-producing bioreactor since 1980; a strain of this fungus named RUT-C30 was characterized by high level of cellulase production and, nowadays, is still exploited for the industrial production of cellulolytic enzymes such as cellobiohydrolases, endo- and exo-glucanases (Peterson and Nevalainen, 2012). The main weakness of *T. reesei* resides in the low level of secreted β-glucosidase, the enzyme responsible for the last step of cellulose degradation, i.e., the cleavage of cellobiose in two D-glucose units. Notably, cellobiose inhibits both endoglucanases and cellobiohydrolases by a product inhibition mechanism, thus pointing to the importance of β-glucosidase for an efficient enzymatic hydrolysis at industrial scale (Sørensen et al., 2013). In this regard, enzymatic blends from *T. reesei* often require supplementation with exogenous β-glucosidases. Important sources of pectinases, xylanases, α-amilases and β-glucosidases are fungi belonging to the genus Aspergillus. Amongst them, *Aspergillus niger* plays a prominent role in the industrial production of pectinases and cellobiase, i.e., a type of β-glucosidase that specifically cleaves cellobiose (Singh et al., 1990; Reginatto et al., 2017). Expression of cellulases, pectinases and α-amylases can be obtained by bacteria using submerged and solid-state fermentation as well. Amongst the many bacteria used as bioreactor, the most relevant in the production of cellulases and pectinases are *B. licheniformis* and *B. subtilis*. In addition to CWDEs from wild-type bacteria, recombinant CWDEs can be produced by *E. coli* through high cell density fermentation. Differently from other fermentation methods, high cell density fermentation is characterized by the highest operating costs (Ferreira et al., 2018). The advantage of using *E. coli* as bioreactor resides in five main factors: (i) rapid growth cycle, (ii) use of cheap growth media, (iii) capability of reaching high cell density through fermentation, (iv) possibility of using strains with low proteolytic activity, and (v) a thorough knowledge of its physiology and genetics. However, the translational machinery of bacteria cannot introduce post-translational modifications such as the formation of cytoplasmic disulphide bridges or glycosylation. Moreover, the poor ability of Gram-negative bacteria to secrete recombinant proteins in the culture medium implies expensive and time-consuming purification procedures, thus representing additional costs for their large-scale production (Table 2). To overcome this drawback, different approaches to enhance the secretion efficiency of recombinant CWDEs in *E. coli* have been developed. The yield of secreted recombinant cellulases and hemicellulases was increased by fusing the catalytic domain of the CWDE of interest to the CBM from another higher soluble cellulase (Murashima et al., 2003) or by reducing the temperature during expression in order to promote a correct protein folding (Song et al., 2012). Alternatively, expression hosts with higher secretion capabilities can be used such as the Gram-positive bacteria *B. subtilis* (Morello et al., 2008) or *Lactococcus lactis* (Pohl and Harwood, 2010), and yeasts; the latter have higher secretion efficiency and can perform post-translational modifications. Many CWDEs have been successfully expressed as secreted proteins in *Saccharomyces cerevisiae* (Margolles-Clark et al., 1996; Haan et al., 2007, 2013; Van Zyl et al., 2010; Zhou et al., 2015), *Kluyveromyces lactis* and *Pichia pastoris* (Bey et al., 2011; Chen et al., 2011; Akbarzadeh et al., 2014; Table 2). Compared to other yeasts, *P. pastoris* and *K. lactis* glycosylate the secreted proteins at lesser extent, reducing the side-effects of unwanted hyperglycosylation. Yeast has been genetically improved to increase CWDE expression levels by the use of synthetic (Blazeck et al., 2013) and constitutive promoters (Haan et al., 2007; Wang et al., 2012), codon-optimized genes (Akcapinar et al., 2011) and selection of multicopy transformants (Yamada et al., 2011; Mellitzer et al., 2012).

### Plant and Microalgae as CWDE-Producing Biofactories

Plant expression of CWDEs can be a valid alternative to microbial-based bio-factories. Plants are characterized by a high [productivity/production cost] ratio and consume atmospheric CO_2_ through photosynthesis, thus representing an eco-friendly expression system. Moreover, plants have GRAS status and are suitable for the production of high-value compounds at affordable costs. However, plant expression of CWDEs may have side-effects since CWDEs from lignocellulolytic fungi and bacteria are well-known pathogenicity factors whose accumulation can damage plant tissue and produce toxic effects (Benedetti et al., 2019a). Moreover, CWDEs can trigger plant defense responses independently of their enzyme activity, being recognized as dangerous non-host molecules through specific recognition mechanisms mediated by plant pattern recognition receptors (PRRs) at the apoplast/outer membrane interface (Poinssot et al., 2003; Ma et al., 2015; Zipfel, 2014; Choi and Klessig, 2016). In general, the expression of pectinases and cellulases in plants can be more insidious with respect to hemicellulases (Benedetti et al., 2015; Castiglia et al., 2016). It is worth mentioning that cell wall fragments derived from the partial breakdown of pectin and cellulose are powerful inducers of plant defense responses (Souza et al., 2017; De Lorenzo et al., 2019). Constitutively activated immunity may lead to detrimental effects due to the growth-defense trade off (Huot et al., 2014; Benedetti et al., 2015), resulting in impaired growth and eventually lethality. Recently, an enzymatic machinery capable of inactivating the elicitor activity of these cell wall derived fragments, namely oligogalacturonides and cellodextrins, has been identified (Benedetti et al., 2018b; Locci et al., 2019). Moreover, gene silencing events may also occur: the nuclear expression of the endoglucanase EGII from *Acidothermus cellulolyticus* in transgenic rice, for example, is suppressed during development from seedling to mature plant, resembling a self-defense mechanism (Li et al., 2019). Gene silencing is a well-known epigenetic mechanism in land plants that undermines the use of transgenic crops for field applications. Different plant expression strategies can be adopted to prevent these undesired event, such as compartmentalized expression and storage, inducible gene expression, expression of CWDEs with inducible activity (i.e., CWDEs with extreme pH- and temperature-dependent activities), use of hosts that are less sensitive to CWDEs (e.g., microalgae) or a combination of these strategies (e.g., chloroplast expression of hyperthermophilic CWDEs) (Benedetti et al., 2019a). The chloroplast expression of CWDEs in tobacco plants has several advantages: (i) high yields of recombinant protein since, unlike nuclear expression, chloroplast expression is not affected by gene silencing events, (ii) easier biocontainment of transgenes since the plastome is maternally inherited, thereby avoiding dispersion through the pollen by vertical gene transfer (Daniell, 2007; Adem et al., 2017), and (iii) the chloroplast expression of CWDEs in tobacco is well-documented, with a recent demonstration that CWDE production may be achieved in field condition without loss in enzyme yield for at least three consecutive growth cycles (Schmidt et al., 2019). Moreover, the chloroplast translational machinery catalyzes the formation of disulphide bridges, enabling the expression of eukaryotic CWDEs in their active form with no need of subsequent renaturation procedures (Taunt et al., 2018). However, the chloroplast system is not suitable for expressing cellulases that require glycosylation for proper activity and stability (Greene et al., 2015), although this trait could be favorable in other cases to avoid the undesired glycosylation of recombinant proteins. Tobacco can be grown in field with a production cost of 2€ kg DW^–1^ and an annual productivity of about 8.1 t (ha^∗^y)^–1^ considering three growth cycle per year as the best achievable for tobacco plants under optimal latitude condition (45°N) (Maksymowicz and Palmer, 1997; Foreman, 2006; Faè et al., 2017; Schmidt et al., 2019; Table 2). Even if a high-density cultivation method has been reported for tobacco plants, transgenic plants expressing CWDEs have never been cultivated with this technology (Scott and Warren, 2012). Enzyme yields from transplastomic tobacco are very encouraging, on the other hand plants need arable land for their growth and longer times to produce significant amounts of recombinant proteins compared to microbes. In this regard, microalgae may represent a good alternative since they are characterized by faster growth rates and the capability of growing at higher CO_2_ concentration than land-plants. Moreover, microalgae can be cultivated in closed growth systems, i.e., photobioreactors, that can occupy waste lands, avoiding the subtraction of arable lands to the agri-food sector (Slade and Bauen, 2013; Tredici et al., 2016; Clippinger and Davis, 2019). Photobioreactors are closed environments where microalgae are exposed to continuous mixing, air supplementation, controlled light and temperature; it comes that algal productivity increases in photobioreactors as well as the production cost. Here, the productivity of microalgae, expressed as biomass produced per year, can reach 60 t (y^∗^ha)^–1^, with a production cost of 3.2 to 3.8 € kg DW^–1^ (Slade and Bauen, 2013; Tredici et al., 2016; Table 2). The production cost of photobioreactor-grown microalgae varies based on three main parameters: (i) the microalgal species of interest, (ii) the size, and (iii) the type of the photobioreactor used for the cultivation (Clippinger and Davis, 2019). The yield of CWDEs in the chloroplast of *Chlamydomonas reinhardtii*, i.e., the microalgae used as model organism, reached lower level compared to that of transplastomic tobacco plants (Faè et al., 2017; Richter et al., 2018); therefore, further optimization is still required for improving the expression stability and protein yield of CWDEs from microalgal-based biofactories (Benedetti et al., 2018a). However, it is worth noting that microalgae culturing may combine the expression of recombinant proteins to the production of valuable biomass (Benedetti et al., 2018a).

## Yield and Cost Analysis of Enzymes From Different Cwde-Producing Biofactories

In this paragraph, the production costs of several CWDEs and productivity of different CWDE-producing biofactories are summarized in order to highlight strengths and weaknesses of each expression system. The limited number of established scientific reports concerning industrial CWDE-production reflects the need of a stronger integration of basic science with applied research. In the techno-economic analyses here reported (Zhuang et al., 2007; Machado de Castro et al., 2010; Humbird et al., 2011; Klein-Marcuschamer et al., 2011; Wilken and Nikolov, 2011; Ferreira et al., 2018), final values were obtained by projecting the results and production costs derived from pilot-scale experiments to large-scale through different simulation software. The production costs are the same as those indicated in the manuscript from which they have been extrapolated, i.e., without normalizing the inflation rate over the years after the publication date. Conversion from United States $ in € was obtained by applying 0.9 as conversion factor. For the production analysis in tobacco plants, dry weight (DW) was considered as 10% fresh weight of the plant biomass. Enzyme activity of plant CWDEs was expressed in Units, i.e., the amount of the enzyme that catalyzes the conversion of one micromole of substrate per minute (μmol min^–1^). Lysozyme activity was expressed in Shugar Units as described in Chiang et al. (1993) and Huang et al. (2002b). All the production costs and productivities as obtained from different CWDE-expressing bio-factories are summarized in Table 3.

**TABLE 3:** Estimation of potential annual productivity and production costs of CWDEs obtained from different bio-factories.

CWDE	Organism	Production method	Annual enzyme productivity t (m^3^*y)^–1^ t (ha*y)^–^^1^	Production Cost		Product value	References

				€ kg DW^–^^1^	U €^–^^1^		
Cellulase	*T. reesei*	SF	2.6	3.82	ND	LOW-VALUE	Humbird et al., 2011
			ND	9.12			Klein-Marcuschamer et al., 2011
	*C. thermocellum*			36.3			Zhuang et al., 2007
		SSF		14.1			
	*N. tabacum* (recombinant)	C	1.13*	14.3*	0.06x10^5^*		Faè et al., 2017/*This review*
			0.32*	50*	ND		Schmidt et al., 2019/*This review*
			0.81*	20*	246x10^5^*		Verma et al., 2010/*This review*
β-glucosidase	*N. tabacum* (recombinant)		0.46*	35*	73x10^5^*		Castiglia et al., 2016/*This review*
	*E. coli* (recombinant)	HDF	0.88	284	ND		Ferreira et al., 2018
Xylanase	*N. tabacum* (recombinant)	C	0.12*	132*	8.1x10^5^*		Castiglia et al., 2016/*This review*
	*A. awamori*	SSF	ND	9.36	0.26x10^5^		Machado de Castro et al., 2010
Pectate Lyase	*N. tabacum* (recombinant)	C	0.85*	18.9*	1.3x10^5^*		Verma et al., 2010/*This review*
			1.14*	14.2*	1.6x10^5^*		
EWL	*G. gallus*	hen egg-white	NA	326*	21.5x10^7^*		Chiang et al., 1993/*This review*
HUL	*O. sativa* (recombinant)	RG	0.048*	150*	133x10^7^*		Huang et al., 2002a,b/*This review*
EWL	*K. lactis* (recombinant)	HDF+P	ND	NA	0.14 x10^7^*	HIGH-VALUE	Huang and Demirci, 2009
	egg-white	egg-white + P	NA	479*+1845	3x10^7^*		Wilken and Nikolov, 2011/*This review*
HUL	RG	RG + P		273*+1971	8.9x10^7^*		

Values reported in the table represent the productivity and production costs of several CWDEs obtained by different biofactories. For data normalization, dry weight (DW) was considered as 10% fresh weight of the tobacco plant biomass. *, values calculated from the available data in the original manuscript. ND, not determined; NA, not applicable. [C, chloroplast expression; EWL, egg-white lysozyme; HDF, high-cell density fermentation; HUL, human lysozyme; P, extraction and purification; RG, rice grains; SF, submerged fermentation; SSF, solid-state fermentation].

### Yield and Cost Analysis of Microbial CWDE-Producing Biofactory

*Trichoderma reesei* is a filamentous fungus employed as cellulase-producing bioreactor secreting more than twenty different cellulolytic enzymes during fermentation. The cultivation of *T. reesei* can be carried out by submerged fermentation using both glucose and steam-exploded poplar as substrates for inducing the secretion of cellulases. By submerged fermentation, *T. reesei* produced up to 2.6 t (y^∗^m^3^)^–1^ of cellulases (Humbird et al., 2011), with a production cost comprised between 3.82 and 9.12 € kg^–1^ (Humbird et al., 2011; Klein-Marcuschamer et al., 2011). It is worth noting that the commercial cellulase-based products are not only composed by cellulase(s). In general, other ingredients added on purpose and contaminants also contribute to the final weight of the cellulolytic blend. For example, some cellulolytic blends are composed by 15% (w/v) of cellulases and 20% (w/v) of glucose, the latter used as stabilizer to prolong the shelf-life of the enzymes (Rodrigues et al., 2015). By submerged fermentation, *A. niger* produced pectinases up to 9400 Units L^–1^, with an enzymatic yield of about 7.7 ^∗^10^5^ Units per kg DW mycelium (Reginatto et al., 2017). A techno-economic analysis on the production of xylanases and α-amylases by *A. awamori* is described in Machado de Castro et al. (2010). In this study, the large-scale production of α-amylases and xylanases and, at lower extent, of cellulases, was carried out by solid-state fermentation using babassu cake as substrate. The highest levels of production were reached upon 6–7 days of solid-state fermentation with values of 250.000- and 85.000-Units kg^–1^ DW for xylanase and amylase activity, respectively. With respect to other filamentous fungi, *Aspergillus awamori* is characterized by the GRAS designation, positively impacting the production cost of the enzymes because the fermented substrate can be sold as feed, thus reducing the total costs of the entire process. According to this possibility, the initial production cost of the CWDE-based product (30.74 € kg DW^–1^) could be reduced to 9.36 € kg DW^–1^ upon selling the fermented babassu cake, reaching the same production cost of enzymatic blends from *T. reesei*. Production of a recombinant β-glucosidase in *E. coli* by high cell density fermentation reached a productivity of 0.88 t (y m^3^)^–1^ and a production cost of 284 € Kg^–1^ using a fermenter of 100 m^3^. At the end of the process, the β-glucosidase was available in a concentrated form (15 g enzyme L^–1^) and ready to be supplemented in cellulolytic blends. Notably, the addition of β-glucosidase in cellulolytic blends in the ratio 0.9:10 [w_β –glucosidase_: w_*cellulases*_] would increase the final price of the enzymatic blend up to 137% (Ferreira et al., 2018). A techno-economic analysis concerning the production of thermostable cellulases by thermophilic bacteria was described in Zhuang et al. (2007). Here, the thermostable cellulases were produced by *Clostridium thermocellum*, a thermophilic bacterium with optimal growth at 60°C, using both the submerged and solid-state fermentation. The main characteristic of this thermophilic bacterium resides in the capability of producing cellulosomes, multi-enzyme complexes characterized by more than twenty different catalytic domains (Gold and Martin, 2007). Submerged and solid-state fermentation allowed the production of cellulases with a cost ranging from 14.1 to 36.3 € Kg^–1^ for those obtained by solid-state and submerged fermentation, respectively. *B. subtilis* is also used as a pectinase-producer organism. Submerged fermentation of *B. subtilis* reached a maximal yield of pectinolytic activity around 66100 Units L^–1^ (Oumer and Abate, 2018), about 7-times higher than that obtained from *A. niger* by using the same fermentation method (Reginatto et al., 2017); to date, techno-economic assessments of pectinase-expressing bio-factories have not been reported despite the high number of studies concerning the production of pectinases through different fermentation procedures (Garg et al., 2016).

### Yield and Cost Analysis of Plant CWDE-Producing Biofactory

Cell wall degrading enzymes production is generally confined to the chloroplast, thus avoiding the interaction with both PRRs and cell wall polysaccharides and preventing the formation of oligosaccharidic elicitors. Nonetheless, chloroplast expression of CWDEs preserved plant fitness at variable extents (Verma et al., 2010; Castiglia et al., 2016; Schmidt et al., 2019). Chloroplast expression of the hyper-thermostable cellulase from *Sulfolobus solfataricus* resulted in tobacco plants with stunted growth and pale-green phenotype despite the expression was compartmentalized and the enzyme activity was inducible by high temperature (Castiglia et al., 2016). Notably, transplastomic tobacco expressing the β-glucosidase from *Pyrococcus furiosus* accumulated 14.5 ^∗^ 10^6^ Units kg DW^–1^ plant biomass and a theoretical enzyme yield of 57 g kg DW^–1^ in accordance with the specific activity of the enzyme (255 Units mg^–1^). In this case, the enzyme productivity was 0.46 t (ha^∗^y)^–1^ while the production cost of the enzyme was 35 € kg^–1^ in the tobacco leaf wet basis. Surprisingly, the specific activity of β-glucosidase from tobacco chloroplast was higher than that reported for the native enzyme from *P. furiosus*, i.e., 750 *vs* 446 Units mg^–1^ as determined at 90°C using *p-*nitrophenyl-glucoside as substrate (Kengen et al., 1993; Castiglia et al., 2016). However, the transplastomic expression of β-glucosidase from *P. furiosus* affected the plant development at variable extent, resulting in both dwarf and wild-type like plants. Similar yield was obtained for the xylanase from *Alicyclobacillus acidocaldarius*; in this case, plant accumulated 1.6 ^∗^ 10^6^ Units kg DW^–1^ plant biomass and a theoretical enzyme yield of 15.2 g kg DW^–1^ in accordance with the specific activity of the enzyme (105 Units mg^–1^). Here, the enzyme productivity was 0.12 t (ha^∗^y)^–1^ while the production cost of the xylanase was 132 € kg^–1^ in the tobacco leaf wet basis. In Faè et al. (2017), transplastomic tobacco plants accumulated 140 g kg DW^–1^ of the cellulase CelK1 from Paenibacillus; notably, plants grew without morphological defect. The enzyme productivity was 1.13 t (ha^∗^y)^–1^, while the enzyme cost was 14.3 € kg^–1^ in the tobacco leaf wet basis. However, the enzyme activity in the leaf tissue was very low (12^∗^10^3^ Units kg DW^–1^) indicating that CelK1 was not very active, with a theoretical specific activity of 0.08 Units mg^–1^. A further study on the production of CWDEs from transplastomic tobacco plants in field-grown condition is described in Schmidt et al. (2019). According to this study, plants stably accumulated the thermostable endoglucanase Cel6A from *Thermobifida fusca* over different growth cycles reaching a maximum yield of 40 g DW^–1^ plant biomass. The enzyme productivity was 0.32 t (ha^∗^y)^–1^, while the production cost of the enzyme was 50 € kg^–1^ in the tobacco leaf wet basis. A comparison of activity would also be necessary, since data on Cel6A activity are not reported in this study. Healthy tobacco plants that accumulated high yield of active endoglucanase and two different pectin lyases are described in Verma et al. (2010). The enzyme productivities ranged from 0.81 to 1.14 t (ha^∗^y)^–1^ for the endoglucanase celD from *C. thermocellum* and pectin lyase PelD from *Erwinia carotovora*, respectively, with an enzyme production cost comprised between 14.2 and 20 € kg^–1^ in the tobacco leaf wet basis.

All these results taken together suggest that plant-CWDE interaction is not easily predictable and therefore, it should be evaluated case by case. As an alternative, photosynthetic hosts less sensitive to plant CWDE could be used, such as the microalga *C. reinhardtii*. Transplastomic expression of endoglucanases has been obtained in *C. reinhardtii* although the enzyme yield was lower than that from transplastomic tobacco plants (Faè et al., 2017; Richter et al., 2018). The lack of a clear phytotoxic effect in *C. reinhardtii* toward plant CWDEs could be ascribed to the algal cell wall composition, mainly protein rather than polysaccharidic (Imam et al., 1985), along with a different host-microbe coevolutionary adaptation. In terms of yield, the chloroplast of *C. reinhardtii* may accumulate recombinant protein up to at most 5% (w/w) of the total soluble proteins (Manuell et al., 2007), corresponding to 1.2 % (w/w) of the algal biomass (Rasala et al., 2010). Cultivation of microalgae can be achieved at high productivity in closed growth systems such as photobioreactors; amongst the several techno-economic analyses performed on such topics, the best-case scenarios were reported by Slade and Bauen (2013) and Tredici et al. (2016). Considering these parameters, *C. reinhardtii* may theoretically produce a hypothetical CWDE up to 12 g kg^–1^ DW of microalgal biomass, with an enzyme productivity of 0.72 t (ha^∗^y)^–1^ and enzyme production costs ranging from 267 to 317 € kg^–1^ in the algal biomass wet basis.

### Case Study: Lysozyme, a High Value CWDE With Many Industrial Applications

Lysozyme isoforms from hen egg-white and human are the most relevant in biotechnological field; human lysozyme (HUL) is preferable over egg-white lysozyme (EWL) due to the higher specific activity (HUL: 201526 Units mg^–1^ *vs* EWL: 70400 Units mg^–1^) (Chiang et al., 1993; Huang et al., 2002b) and to the “safer” designation since HUL, unlike EWL, does not cause allergic reactions in humans (Ercan and Demirci, 2016). Notably, the use of lysozyme in medicine implies further purification procedures and, therefore, higher production costs compared to other CWDEs with application as low-value products (e.g., biofuels, paper industry). Due to its hydrolysing activity toward the cell wall of bacteria and filamentous fungi, expression in such organisms is unfeasible. Lysozyme is commonly obtained from hen egg-white upon extraction and purification. The amount of lysozyme is around 0.15 g per egg (Abeyrathne et al., 2013); given that the average weight of an egg is 65 g and that the production cost of a single egg is 0.05 € in a conventional hen-housing system, one kilogram of eggs contains about 2.3 g of lysozyme with a production cost of 0.75 € (Matthews and Sumner, 2015). Based on these estimations, the production cost of EWL is around 326 € kg^–1^ in the egg wet basis. Wilken and Nikolov estimated that the purification procedure for obtaining EWL with a purity greater than 94% had an approximate cost of 1845 € kg^–1^ (Wilken and Nikolov, 2011); such procedure was characterized by a purification yield of 68% indicating that about 600 kilograms of eggs are required to obtain 1 kg of pure lysozyme. According to this scenario, the production cost of a high-purity lysozyme preparation from egg-white is 2295 € kg^–1^ (3 × 10^7^ Units €^–1^). However, many lysozyme-based products such as those used in food processing are characterized by lower costs (expressed as € kg^–1^ product) because the real lysozyme content does not correspond to the weight of the product. In these cases, customers should always refer to the Units per gram of product rather than to the sole weight. In this regard, it is worth noting that the enzymatic Units of lysozyme can be defined through diverse methods, making the comparison between different lysozyme-based products rather uncertain.

In addition to the hen egg-white, other important sources of lysozyme are recombinant yeast and rice. Lysozyme can be purified upon production by *Kluiveromyces lactis* through high-cell density fermentation with higher costs, i.e., 0.14 × 10^7^ Units €^–1^ (Huang and Demirci, 2009). Importantly, the use of yeasts for expressing CWDEs with potential applications in the biomedical field requires great caution since the secreted CWDEs could be hyper-glycosylated. Glycosylated proteins from yeast-based biofactory cannot be used in food processing and medicine due to the possibility of triggering allergic reactions in humans. Fortunately, the amino acid sequence of lysozyme is not characterized by the presence of potential *N*-glycosylation sites. The most relevant bio-factory of HUL is transgenic rice (*Oryza sativa*). The expression level of lysozyme in the rice grain reaches 6 g kg^–1^ DW and therefore, 1 kilogram of recombinant lysozyme is contained in about 167 kg of rice grains (Huang et al., 2002a). Bearing in mind that the annual rice productivity can reach about 8 t (ha^∗^y)^–1^ under the best climate and irrigation conditions^2^ and the production cost of recombinant grain flour is 0.9 € kg^–1^ DW, i.e., five-fold higher than the common grain flour (Wilken and Nikolov, 2011), then the annual productivity of HUL from transgenic rice is around 0.048 t (ha^∗^y)^–1^ with a production cost of 150 € kg^–1^ in the rice flour wet basis. Wilken and Nikolov estimated that the purification procedure for obtaining HUL from recombinant rice with a purity greater than 91% had an approximate cost of 1971 € kg^–1^. This procedure was characterized by a purification yield of 55% indicating that about 304 kg of rice grains are required to obtain 1 kg of pure lysozyme. According to this information, the production cost of pure HUL from recombinant rice is 2244 € kg^–1^ (8.9 × 10^7^ Units €^–1^).

## Conclusion

To date, the enzymatic conversion of cell-wall materials into fermentable sugars is characterized by low-efficiency and high operating costs. For enzymatic hydrolysis to be economically feasible in large scale productions, the optimization of CWDE-producing biofactories is necessary to reduce enzymes cost and improve the degradation activity of CWDE-blends. Improvement of degradation can be obtained by including enzymes with novel and/or improved catalytic activities (e.g., CWDEs from Extremophiles) to the existing enzymatic mixtures. Wild-type microbes secrete a wide array of CWDEs during fermentation and are therefore bio-factories of first choice when a mixture of CWDEs is preferable over the production of a single enzyme, with production costs ranging from 4 to 36 € kg^–1^ enzymatic product depending on the microbial species used (Table 3). However, if a single CWDE is required, the heterologous expression can be a better option. Several factors must be considered for establishing heterologous expression of CWDEs, such as the enzyme’s characteristics, possible toxicity or side-effects for the host organism and the target application field, that may or may not require high enzyme purity. Factors such as the secretion ability of yeast or the ability to form disulphide bridges can have a significant impact on the expression of CWDEs (Table 2). On the other hand, yeasts may hyper-glycosylate the recombinant protein with possible alteration of the enzyme activity or possible allergenicity in humans; the latter point is crucial for those CWDEs that are planned to be used in nutraceutical or biomedical fields (Table 2). In this context, chloroplast expression represents a good alternative since plants are GRAS organism. Moreover, the translation machinery of the chloroplast can direct the synthesis of proteins with correct folding and disulphide bridge formation, thus allowing the expression of different categories of CWDEs. Tobacco chloroplast allowed to obtain high amount of different CWDEs with production costs ranging from 14 to 132 € kg^–1^ enzyme, depending on the type of CWDE. As an example, a recombinant β-glucosidase was obtained from transplastomic tobacco plants at a production cost eight times lower than in *E. coli*; however, to produce 0.9 tonnes of β-glucosidase in tobacco may require two years and one hectare of arable land, whereas the same amount may be obtained just in two weeks using a 26 m^3^ fermenter for *E. coli* (Table 3). Taking into account that (i) CWDEs are used to degrade lignocellulosic biomass at industrial scale with a cost-effective enzyme loading from 0.5 to 1.5% (w/w) (application sheets of Cellic CTec2, HTec2-Enzymes, and Cellic CTec3 for hydrolysis of lignocellulosic materials; Herrero Garcia et al., 2019), (ii) the annual production of lignocellulosic waste from agriculture residues is 4.6 billion tonnes (Dahmen et al., 2019), and (iii) the annual production of cellulases and pectinases from transplastomic tobacco can be estimated around 1 tonne CWDE per hectare (Table 3), it comes that 23 to 69 million hectares (1.6 to 4.9% world’s arable lands) would be required to cultivate enough transplastomic tobacco plants for satisfying such enzyme request. Thus, a high product demand may force out of the market plant-based biofactories since arable lands are in competition with the agri-food production. In this perspective, microalgae could represent the missing link between microbial- and plant-based biofactory since they are characterized by a faster growth rate than plants and offer the possibility of combining the expression of recombinant proteins with the production of valuable biomass. However, transgenic expression in microalgae still requires further optimization.

It is worth noting that several CWDEs are perceived as pathogenicity factor by plants and therefore, their expression should be evaluated case by case. On the other hand, plants can express CWDEs that are toxic for microbes, as in the case of human lysozyme that has been produced in transgenic rice. Similarly, since an efficient chitinase-expressing biofactory is still missing, the chloroplast expression of a hyper-thermostable chitinase in tobacco could be advantageous, because unlike what occurs in microorganisms, this type of CWDE is not toxic for plant health.

Special attention must be paid to the quality of the expressed “CWDE/enzymatic product”; in some cases, the recombinant CWDE can display altered activity likely due to post-translational modifications and other issues related to the heterologous expression (Table 2), or the CWDE pool produced by wild type microbes can be characterized by a low enzyme content per kg of product. In all these cases, both the enzyme production costs and enzyme loading in industrial practices must refer to the Enzyme Units rather than the protein amounts (Table 3).

In conclusion, wild-type microbes are the biofactory of first choice to express CWDEs for industrial processes requiring the action of multiple CWDEs (e.g., lignocellulose degradation), while plants are more indicated to express specific CWDEs, especially when the expression in microbial host is challenging and the application field will imply the use of high value products (e.g., food processing, medical field). In this context, microalgae have a great but still unexploited potential.

In the last decades, the potential of different CWDE-expressing biofactories has been widely investigated, contributing to define the present scenario. Thus, the type and the characteristics of the expressed CWDE, the enzyme cost and productivity, the latter in relation with the market demand of the enzyme, are parameters that determine the effectiveness of the selected biofactory.

## Author Contributions

MG, MB, and BM identified patterns and trends in the literature and designed the structure of the review. MG, GG, and MB prepared the tables and figures. All the authors contributed to the search for relevant literature, carried out a critical analysis of the literature, discussed together, and wrote the manuscript. All authors read and approved the final manuscript.

## Conflict of Interest

The authors declare that the research was conducted in the absence of any commercial or financial relationships that could be construed as a potential conflict of interest.

## Footnotes

1 www.cazy.org

2 http://ricepedia.org/rice-as-a-crop/rice-productivity
